# Genetic diversity of horses of the Sargarinsko-Alexeevskaya and Irmen cultures of the Ob-Irtysh region of Western Siberia and their genetic proximity to modern horses of indigenous breeds

**DOI:** 10.18699/vjgb-26-49

**Published:** 2026-05

**Authors:** M.А. Kusliy, A.A. Yurlova, N.V. Vorobyeva, A.A. Proskuryakova, M.A. Demin, S.M. Sitnikov, V.N. Zharonkin, S.S. Onishchenko, A.K. Kasparov, A.E. Tupikin, A.S. Graphodatsky, A.S. Molodtseva, A.A. Tishkin

**Affiliations:** Institute of Molecular and Cellular Biology of the Siberian Branch of the Russian Academy of Sciences, Novosibirsk, Russia; Institute of Molecular and Cellular Biology of the Siberian Branch of the Russian Academy of Sciences, Novosibirsk, Russia; Institute of Molecular and Cellular Biology of the Siberian Branch of the Russian Academy of Sciences, Novosibirsk, Russia; Institute of Molecular and Cellular Biology of the Siberian Branch of the Russian Academy of Sciences, Novosibirsk, Russia; Altai State Pedagogical University, Barnaul, Russia; Autonomous non-profit organization “Altai Archaeological Society”, Barnaul, Russia; Department of Roskomnadzor for the Kemerovo Oblast, Kemerovo, Russia; Committee for the Protection of Cultural Heritage of Kuzbass, Kemerovo, Russia; Institute for the History of Material Culture of the Russian Academy of Sciences, St. Petersburg, Russia; Institute of Chemical Biology and Fundamental Medicine of the Siberian Branch of the Russian Academy of Sciences, Novosibirsk, Russia; Institute of Molecular and Cellular Biology of the Siberian Branch of the Russian Academy of Sciences, Novosibirsk, Russia; Institute of Molecular and Cellular Biology of the Siberian Branch of the Russian Academy of Sciences, Novosibirsk, Russia; Altai State University, Barnaul, Russia

**Keywords:** ancient DNA, mitochondrial genome, phylogenetics, domestic horse, Bronze Age, Sargarinsko-Alexeevskaya culture, rmen culture, древняя ДНК, митохондриальный геном, филогенетика, домашняя лошадь, бронзовый век, саргаринско-алексеевская культура, ирменская культура

## Abstract

Genetic diversity of horses of the Sargarinsko-Alexeevskaya and Irmen cultures of the Ob-Irtysh region of Western Siberia and their genetic proximity to modern horses of indigenous breeds

## Abstract (ru)

Генетическое разнообразие лошадей саргаринско-алексеевской и ирменской культур Западной Сибири и их генетическое родство с современными лошадьми аборигенных пород

## Introduction

In the context of equine genetic research, the Bronze Age (BA) has been less studied than the Iron Age (Keyser-Tracqui et al., 2005; Dawei et al., 2007; Cai et al., 2009; Lei et al., 2009; Cieslak et al., 2010; Benecke et al., 2017; Fages et al., 2019; Vorobieva et al., 2020; Kusliy et al., 2021; Librado et al., 2021; Kusliy, 2023). However, some important data have already been obtained. Regarding the territory of Inner Asia, it has been found that horses of the Sintashta culture, which is associated with the first ritual burials of horses together with parts of chariots with spokes, made a significant contribution to the equine gene pool of many subsequent Central Asian cultures, including the Khereksur and “Deer Stone” culture of Mongolia (Fages et al., 2019) and the Altai Biykenskaya culture (Librado et al., 2021). It should be noted that the Sintashta culture was the earliest stage in the development of the Andronovo cultural and historical community, which spread across the territory of the Southern Urals, the south of Western Siberia, Kazakhstan, and the western part of Central Asia (Zubova et al., 2014).

In this study, we examined the mitogenome diversity in horses of the Irmen and Sargarinsko-Alexeevskaya cultures, widespread in the Ob-Irtysh region of Western Siberia in the Late Bronze Age (Grushin, 2020). Bone materials for genetic analysis had been obtained from the sites of Chekanovsky Log-I (Demin, Sitnikov, 1998), Barsuchikha-IV of the Sargarinsko-Alexeevskaya culture, Kaltyshino V (Kovtun, 2022), Barsuchikha-IV, Gusinaya Lyaga-1 (Demin, 2015) of the Irmen culture. Researchers of some of these sites had noted the important role of horses in various spheres of life of the inhabitants of these sites; for example, the majority of bone remains of domestic animals found at the Gusinaya Lyaga-1 site belonged to horses (Demin, 2015). Materials from both cultures studied were identified at the archaeological sites of Barsuchikha-IV and Gusinaya Lyaga-1, so the researchers classified these complexes as mixed-type sites (Sitnikov, Gel’mel’, 2017). The results of our study highlight differences in the horse maternal genetic lineages of the Sargarinsko- Alexeevskaya and Irmen cultures, which allows us to hypothesize different maternal origins of horses of these cultures and the absence of intensive exchange of horses between them. It is also shown that the horses of mixed sites on the border between these cultures are closer in mitogenomes to the Sargarinsko-Alexeevskaya culture horses. However, this hypothesis requires confirmation based on more sample sets from each culture.

Thus, according to archaeological data (Grigor’ev, 2018; Kovalevsky, 2020), the Andronovo culture made a great contribution to the formation of the Irmen and Sargarinsko- Alexeevskaya cultures. We included samples from Altai and Kazakhstan sites of this culture in our study in order to trace possible continuity. Archaeological data provide no comprehensive answer to questions about other ancestral cultures or the continuity of the cultures under consideration in relation to later cultures of the region (Sitnikov, 2013; Papin et al., 2018; Popova, 2019). In order to strengthen the evidentiary base for resolving these issues, we supplemented our materials with samples of the Early BA Eluninskaya culture, formed in the Forest-Steppe Altai as a result of the migrations of Indo- European tribes to the eastern and southern regions of Eurasia (Kiryushin, 2002; Grushin, 2019), and from synchronous and later sites of the Altai and Mongolia (Tishkin, 2007)

## Materials and methods

Materials studied. Our material included two bone samples of the Eluninskaya culture from Altai (Berezovaya Luka site), two bone samples of the Andronovo culture from Altai and Kazakhstan (Chekanovsky Log-2 site (Altai), Tasty-Bulak site (Kazakhstan)), six bone samples from Altai mixed sites of the Irmen and Sargarinsko-Alexeevskaya cultures (Barsuchikha- IV and Gusinaya Lyaga-1 sites), two bone samples of the Irmen culture from southern Western Siberia (Kaltyshino V sites), four bone samples of the Sargarinsko-Alexeevskaya culture of Altai (Chekanovsky Log-I site), and one bone sample of the Bystryanskaya culture (Manzhikha-2 site (Altai)). Detailed information about the samples studied is given in Supplement 1^1^. Supplement also provides information on radiocarbon dates of samples whose archaeological dating was ambiguous. Radiocarbon dating was performed at the AMS Golden Valley (Novosibirsk, Russia). Accurate dating of a sample is necessary to prevent errors in the cultural assignment of samples.

Supplementary Materials are available in the online version of the paper: https://vavilovj-icg.ru/download/pict-2026-30/appx25.zip


Ancient mitogenome sequencing. All experiments were performed at the Institute of Molecular and Cellular Biology (IMCB SB RAS), Siberian Branch of the Russian Academy of Sciences, in a special ancient DNA laboratory, in accordance with the basic authenticity criteria for ancient DNA research (Willerslev, Cooper, 2005), which presently remain relevant: (1) The work areas for ancient DNA experiments before the PCR stage and after it were separated. (2) Laboratory rooms for experiments with ancient DNA before the PCR stage were equipped with a ventilation system that created elevated pressure; experimenters wore special laboratory suits and two pairs of gloves, the outer one of which was constantly changed; the gloves and all work surfaces were constantly wiped with decontaminants (DNArid (Biomedical Innovations), Dezomax (Maxima LLC)) before, during, and after work; ultraviolet lamps (30 W) and an air sterilizer (recirculator ORB-2N (POZIS)) were also used to decontaminate work surfaces; tubes with samples and reagents were opened only inside laminar-flow cabinets (BAVnp-01-“Laminar-S”-1.5 LORICA (LANSYSTEMS)) with the ventilation turned on. (3) We added negative controls at the stages of isolating ancient DNA and preparing genomic libraries, which we conducted through all processes of the experiment to verify the absence of cross-contamination and foreign contamination in the samples. (4) At the stage of sequencing data analysis, the sizes and base deamination profiles of ancient DNA fragments were evaluated (subsection Statistics and authenticity of sequencing data of the Results and Discussion section). (5) The ancient origin of the samples was proven based on the archaeological context of related materials and direct and indirect radiocarbon dating (Supplement 1).

The preparation of bone samples for DNA extraction and the DNA extraction process itself, as well as the method used to prepare double-stranded, dual-index libraries for sequencing, are described in detail in (Kusliy et al., 2021).

Two rounds of library enrichment were performed using hybridization with biotinylated modern mtDNA of Equus caballus, immobilized on Dynabeads M-280 Streptavidin magnetic particles (Life Technologies, USA). The method reported in (Maricic et al., 2010) was modified as follows: (1) When preparing biotinylated samples, we ligated a doublestranded adapter with a 3′ single ‘T’ nucleotide overhang (5′-CCTGCCTCGGATGTCCTTGAT-3′) to modern horse DNA fragments using a TruSeq Nano DNA Sample Preparation Kit (Illumina) according to manufacturer’s recommendations. (2) Modern horse mitogenome fragment libraries were amplified using biotinylated primers (Biotin-5′-CCTGCC TCGGATGTCCTTGAT-3′). (3) Biotinylated probes were immobilized onto magnetic beads with streptavidin according to the Dynabeads™ Streptavidin Trial protocol using sodium citrate saline (SSC) as binding and washing buffer. (4) The final purification of magnetic particles after the enrichment procedure was carried out in SSC (3 times with 100 μL of 2× SSC at 65 °C for 5 min, 2 times with 100 μL of 0.2× SSC at room temperature). (5) At the final stage of the procedure, 20 cycles of amplification of enriched libraries were conducted. Amplification of the enriched libraries was performed in a volume of 50 μL containing 1x Phusion HF Buffer, 0.2 mM of each dNTP, 1 μМ of each primer for library fragment adapters (SuD Nano DNA Library Prep Kit for Illumina), and 1 U of Phusion DNA polymerase. The PCR program was as follows: 30 s initial denaturation at 95 °C; 20 cycles, each of which included 20 s denaturation at 98 °C, 20 s primer annealing at 65 °C, 20 s elongation at 72°C; and final 5 min elongation at 72 °C

Quantification of the obtained libraries was performed using a Qubit 4 Fluorometer (Invitrogen) and a Qubit dsDNA HS Assay Kit, according to manufacturer’s recommendations. To check for contamination at the stages of DNA isolation, library preparation, and amplification, blanks were used that showed the absence of target DNA during final data analysis. Paired-end sequencing of the enriched libraries was carried out on the MiSeq platform (Illumina, USA) using a MiSeq v2 Reagent Kit (300-cycles, 2x150bp). Library sequencing was performed at Genomics Core Facility (ICBFM SB RAS, Novosibirsk).

Sequence data analysis. Secondary data analysis performed in PALEOMIX BAM Pipeline v1.3.2 (Schubert et al., 2014) is described in detail in (Kusliy et al., 2021).

Phylogenetic analysis. Multiple alignment of the mitogenome consensus sequences was conducted using the MAFFT v1.4.0 multiple sequence alignment program (Katoh et al., 2002) (plugin MAFFT in Geneious Prime v2024.0.4). PartitionFinder v2.1.1 (Lanfear et al., 2012) was used to select the best-fit partitioning schemes and models of molecular evolution for phylogenetic analyses. In the alignment of horse mitochondrial genome sequences, five partitions were identified with the following evolutionary models of nucleotide substitutions: HKY+I for the second codons of protein-coding genes; HKY+G+I for RNA coding genes, first and third codons of protein-coding genes; GTR+G+I for hypervariable regions. The Bayesian phylogenetic tree was constructed with the MrBayes v.3.2.6 program (Ronquist, Huelsenbeck, 2003) using the above partitioning schemes and evolutionary models and the following parameter values: 8 million generations of Markov chain Monte Carlo, sampling frequency 800; the first 25% of trees discarded. The tree visualization was performed with the FigTree v1.4.4. program (“http://tree.bio.ed.ac.uk/ software/figtree/).

Haplotype diversity analysis. The haplotype median joining network and haplogroup map were generated in the PopART population genetics software version 1.7 (Bandelt et al., 1999; Leigh, Bryant, 2015) with standard parameter values

Analysis of genetic differentiation of populations. The FST values, reflecting the measure of population differentiation (10,000 permutations), as well as nucleotide diversity values, were obtained with the Arlequin v3.5.2.2 integrated software package (Excoffier, Lischer, 2010).

Data availability. All bone samples were obtained from the collection in the repository of the Institute of History and International Relations of Altai State University (Barnaul, Russia) (collection manager Dr. Alexey A. Tishkin). The described study complies with all relevant regulations. The GenBank accession numbers of the mitogenome consensus sequences are given in Table.

**Table 1.**
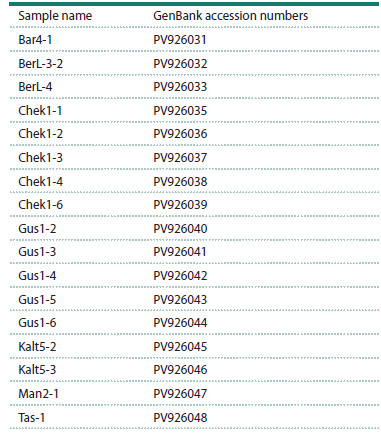
The GenBank accession numbers of the obtained nucleotide sequences

## Results and discussion

Statistics and authenticity of sequencing data. The ancient origin of the obtained sequences is proven by the profiles of nucleotide misincorporation in DNA fragments of the sequencing libraries obtained in the MapDamage v2.2.0 computational framework (Jónsson et al., 2013). The percentage of nucleotide substitutions therein increases towards the ends of the fragments due to the presence of protruding ends in the fragments of ancient DNA (Green et al., 2006; Sawyer et al., 2012). The distribution graphs of the sizes of the sequenced libraries also confirm the data authenticity, since the average fragment size of each library is extremely small (less than 100 bp), which is typical for degraded ancient DNA (Sawyer et al., 2012). The graphs of postmortem damage and fragmentation patterns are shown in Supplement 2. The statistical parameter values of the obtained sequencing data are given in Supplement 3. The frequency of deamination at the ends of DNA fragments, which is on the average 2–3 times higher than in the middle of the fragment (Supplement 3), also points to the ancient origin of the DNA under study

**Fig. 1.**
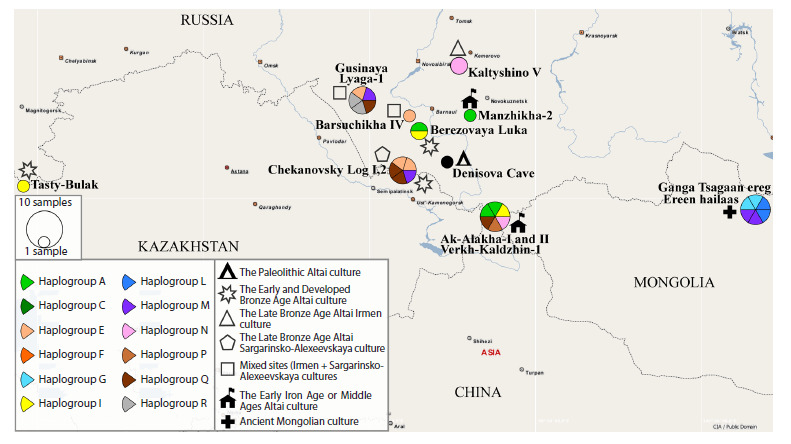
Map of the geographical distribution of haplogroups of ancient and medieval horses in Inner Asia and adjacent territories studied here and earlier. The circle size is proportional to the number of haplotypes obtained. The sector colors highlight different haplogroups. The symbols show the belonging of the sites to a time range or archaeological culture. The legend is shown in the inset.

Haplotyping and haplogroup diversity assessment among the ancient horses studied. We compared the consensus sequences of the mitogenomes of the ancient horses studied here and in previous works (Vorobieva et al., 2020; Kusliy et al., 2021) with each other, determined their characteristic nucleotide variants relative to the reference sequence and assigned them to previously determined haplogroups (Achilli et al., 2012). Based on this data, a map of the geographical distribution of these mitochondrial haplogroups in Inner and Central Asia in ancient times was constructed. The map is shown in Figure 1.

As can be seen from the figure, the haplotype of the Paleolithic Altai horse is not found among the studied ancient or medieval horses in Inner Asia and adjacent territories

The most ancient haplotypes of domestic horses, namely those of the Early and Developed Bronze Age (Andronovo culture (Chekanovsky Log-2, purple sector; and Tasty-Bulak sites) and Eluninskaya culture (Berezovaya Luka site)), belong to haplogroups A, I, and M. These ancient haplogroups were also found in the Altai Krai (Irmen + Sargarinsko-Alexeevskaya cultures) and Mongolia horse groups in the Late Bronze Age, and in the mountain and steppe Altai in the Early Iron Age, which may point to a certain genetic continuity of horses of the corresponding cultures

The figure also shows significant differences in the composition of haplogroups between the ancient horses of Altai and Mongolia, which share only haplogroup M, present in the Andronovo group. In contrast to the small overlap in mitochondrial genetic diversity shown between ancient horses of the Mongolia and Altai regions, modern horses of these regions show a much greater degree of genetic closeness (Kusliy et al., 2023). This observation is most likely indicative of a common origin and the absence of close contacts between horses associated with the cultures of these territories after the Developed Bronze Age.

**Fig. 2.**
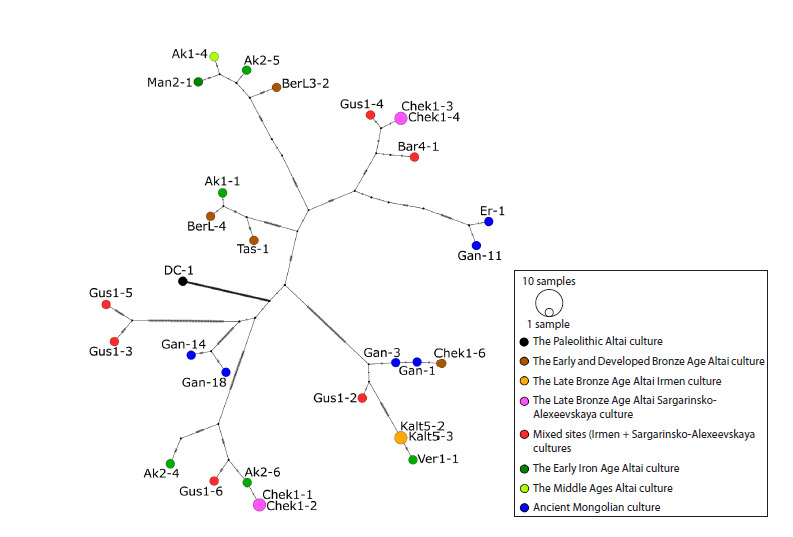
Median joining haplotype network of studied ancient and medieval horses in Inner Asia and adjacent territories. The colors of the circles are associated with different semantic groups of horses (according to historical and cultural periods, belonging to a specific culture or region). The legend is shown in the inset. Lines on the branches of the network represent genetic variants that distinguish the ancestral haplotype from younger ones

Turning attention to the Irmen and Sargarinsko-Alexeevskaya cultures of the Late Bronze Age, which are in the focus of this study, we note that the mixed-type sites in the northern Altai Krai are close in haplogroup diversity to the sites of the Sargarinsko-Alexeevskaya culture of the southern Altai Krai, and they both differ greatly in these indicators from the sites of the Irmen culture, located in the north-eastern direction. As can be seen from the map, the mitochondrial haplogroups of horses from the Irmen sites coincide only with the haplogroups of horses from the Pazyryk (Verkh-Kaldzhin-1) sites of Early Iron Age, located in the south of the Altai Republic. Since, according to archaeological data, the Irmen culture did not contribute to the formation of the Pazyryk culture, this closeness of haplogroups most likely traces the common origin of the horses of these cultures but not continuity between them. In order to determine the differences between the mitogenome haplotypes of the studied ancient and medieval horses of Inner Asia and the adjacent territories from each other, we built a median joining haplotype network (Fig. 2). 

As can be seen from the network, the most ancient haplotypes of domestic horses in the sample (Early and Developed Bronze Age, brown) are closest to the haplotypes of Mongolian horses of the Late Bronze Age (blue) and haplotypes of Altai horses of Early Iron Age (green). The constructed network shows the proximity of horse haplotypes of the Sargarinsko- Alexeevskaya culture and mixed sites of this and Irmen cultures (pink and red, respectively) and their remoteness from horse haplotypes of the Irmen culture, which cluster together with haplotypes of Early Iron Age Pazyryk horses from the territory of the Altai Mountains. The resulting haplotype network also visualizes the differences between the haplotypes of the ancient Mongolian horses under study and most of the other haplotypes of the sample and the similarity of the Bronze Age Mongolian horses to the Andronovo horse, which is most likely related to their origin. The described figure shows a more detailed history of the studied ancient horse haplotypes, whose key features are outlined above.

Our analysis of population differentiation in the culture groups of Sargarinsko-Alexeevskaya (samples Chek1-1, Chek1-2, Chek1-3, Chek1-4) and Irmen (samples Kalt5-2, Kalt5-3) and in the group of mixed sites of these two cultures (samples Bar4-1, Gus1-2, Gus1-3, Gus1-4, Gus1-5, Gus1-6) revealed a high degree of differentiation between the Mixed and Irmen groups (FST = 0.24, p-value > 0.05) and a very high degree of differentiation (FST = 0.52, p-value > 0.05) between the Sargarinsko-Alexeevskaya and Irmen groups. Their FST analysis also showed an insignificant degree of differentiation between the Mixed and Sargarinsko-Alexeevskaya groups. However, since the sample sizes were small, and the p-values were unreliable ( p-value > 0.05), this can only be considered an additional evidence.

**Fig. 3.**
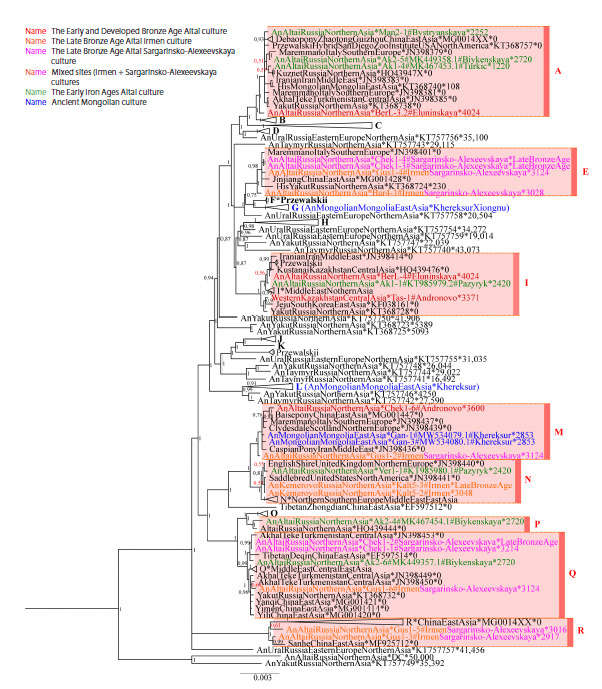
Bayesian phylogenetic tree of ancient, medieval and modern horses from different regions of the world, constructed on the grounds of mitogenome sequences. The names of the samples consist of three parts, separated by an asterisk: (1) the geographical origin of the sample (the An prefix at the beginning denotes ancient and medieval samples, and the His prefix indicates historical horses); (2) registration number of the GenBank database or name of the sample and culture; (3) age of the sample. Letters A–R to the right of the clades correspond to the names of equine mitochondrial genome haplogroups according to the classification by Achilli et al. (2012). The colors of the sample names are associated with different semantic groups of horses (according to historical and cultural periods, belonging to a specific culture and/or region); the correspondence is described at the upper left of the figure. For better clarity, some clades of the tree have been collapsed. The Bayesian posterior probability of the tree topology is shown as numbers next to the tree nodes (probability less than 0.7 (confidence level) is highlighted in red). The numeral under the horizontal scale bar at the bottom of the figure indicates the number of nucleotide substitutions related to a segment of equal length on a tree branch.

Phylogeographic reconstructions. In order to see a more detailed picture of the relationships between haplotypes within haplogroups and to trace phylogenetic relationships to the present, we constructed a Bayesian phylogenetic tree based on mitogenome sequences of ancient, medieval, and modern horses from different regions of the world, including our samples and previously published ones (GenBank sequence database). The constructed tree is shown in Figure 3.

In the above discussion of phylogeographic reconstructions, only clades with high Bayesian posterior node probabilities are considered in the analysis of reliable phylogenetic relationships

The constructed phylogenetic tree shows that haplogroup I represents the intersection of haplogroups of the Eluninskaya and Andronovo cultures of the Bronze Age. One of the Eluninskaya horses we studied is located basal to the clade of all other domestic horses of haplogroup A, which supports the origin of horses of this haplogroup from horses of this culture. However, since the Andronovo horses not included into our study could also occupy a basal position within this haplogroup, we cannot rule out the Andronovo origin. The Eluninskaya culture is known to form earlier (Early Bronze Age) than the Andronovo one (Developed Bronze Age). Most of the animal bones found in the Berezovaya Luka settlement (samples BerL-3.2, BerL-4) belong to domestic species (Kiryushin et al., 2011), but it has not been shown whether the horses of Berezovaya Luka site were domesticated or wild (hunted). Our data (Fig. 3, haplogroup I) clearly indicate that some horses of this settlement were domesticated, because the corresponding haplotypes are located within the clade of modern domestic horses. Since the development of animal husbandry in the Altai steppe zone is primarily associated with the bearers of the Eluninskaya culture, who migrated there at the beginning of the Early Bronze Age (Kiryushin et al., 2012), the South Siberian origin of some ancient domestic horses of Asia remains questionable. However, since the Eluninskaya and Andronovo cultures overlapped in time, and horses of the Andronovo culture spread very quickly to adjacent territories (Koryakova, Epimakhov, 2007; Lindner, 2020; Epimakhov, 2020), the question of local domestication or Andronovo origin of the Eluninskaya culture horses will remain relevant until whole genome data on horses of these cultures are obtained. Based on our data, we conclude that some domestic horses from both cultures are genetically very close. It should also be noted that, according to our results, the haplogroups of the Eluninskaya culture horses were found only among horses of Early Iron Age from the south of the Altai Republic (Biykenskaya, Pazyryk, Turkic cultures), and the haplogroups of the Andronovo culture were widespread not only among the Pazyryk horses of Altai, but also among the horses of the Late Bronze Age from the Ob-Irtysh region of Western Siberia (Irmen + Sargarinsko-Alexeevskaya cultures) and Mongolia (the Khereksur and “Deer Stone” culture). The closeness of the haplotypes of horses of the Khereksur and “Deer Stone” culture of Mongolia and the Andronovo culture, detected by us for the first time on the basis of mitogenome data, is most likely associated with continuity between the horses of these cultures. A horse of the mixed site of the Irmen and Sargarinsko-Alexeevskaya cultures, which occupied the basal position in clade M, is also quite close to the mitochondrial haplotypes under consideration (samples Gan-1, Gan-3, Chek1-6). The closeness between the haplotypes of domestic horses of the Andronovo culture, from which most domestic horses of Asia originated, and horses of later cultures of Mongolia and Siberia more likely points to their common origin from horses of the Andronovo cultural–historical community. The intersection of the mitogenome gene pools of horses of cultures later than Andronovo in Siberia and Mongolia is more likely to point to close contacts between archaeological cultures of these regions.

Within haplogroup I, the BerL-4 horse of the Eluninskaya culture, one of the most ancient in our sample occupies the closest position to horses of the Kustanai local breed of Kazakhstan and the indigenous Iranian breed. This observation looks consistent with the migration of part of the population of the Eluninskaya culture to the west after the arrival of the Andronovo population to their territory (Grushin, 2018).

Within haplogroup E (Fig. 3), which turned out to be one of the key haplogroups of the Sargarinsko-Alexeevskaya culture and mixed sites (Irmen+Sargarinsko-Alexeevskaya), the domestic horses of the latter-listed cultures we studied are located next to modern horses of the indigenous breeds of Italy (Maremmano), China (Jinjiang), and historical Yakutian horses. The last of these horse breeds migrated to Yakutia from southward regions together with the Yakut people several centuries ago (Librado et al., 2015). The genetic closeness we identified between the ancient domestic horses of the mixed sites we studied and the Yakut horses is also noticeable within haplogroup Q. There is evidence that the Maremmano horse breed was formed in the 8th century BCE by Etruscans (Giontella et al., 2020; Vernesi et al., 2004), an ancient people primarily of South European origin (Ghirotto et al., 2013; Vernesi et al., 2004). Previous studies have shown maternal genetic closeness between modern Maremmano horses and domestic horses of the Altai Biykenskaya culture of Early Iron Age (Vorobieva et al., 2020). The Jinjiang horse is a breed indigenous to Central and Southeast China; it formed at the end of the 1st millennium CE and developed with a small genetic infusion from foreign breeds (Ma et al., 2019). The similarity of mitochondrial haplotypes of horses of the Sargarinsko-Alexeevskaya culture, mixed sites, and modern domestic horses of indigenous breeds from different regions of China (Yanqi, Yimen, Yili, Sanhe) can also be traced within the Q and R haplogroups. The basal position of one of the horses from the mixed Irmen and Sargarinsko-Alexeevskaya culture site Barsuchikha-IV in relation to the other horses of clade E and clade M indicates that some horses of the Maremmano, Jinjiang, and Yakutian breeds might derive from ancient domestic horses of these cultures, with migration occurring in both the eastern and western directions.

As can be seen from the phylogenetic tree we constructed, the horses of the Sargarinsko-Alexeevskaya culture and the mixed sites of the Irmen and Sargarinsko-Alexeevskaya cultures are also very close in mitochondrial DNA to modern Akhal-Teke horses (haplogroup Q), one of the most ancient riding breeds of Central Asia (Szontagh et al., 2005).

Horses of the Irmen culture were assigned to clade N, which is mainly represented by modern horses of local breeds of Southern and Northern Europe and breeds formed on their basis. Within this haplogroup, horses of the Irmen culture are located in the subclade that also contains horses of Early Iron Age Pazyryk culture in Altai and modern horses of the English Shire breed and the American Saddlebred breeds. The English Shire breed is native to England (Stephens, Splan, 2013), and the American Saddlebred breed was developed on the basis of many riding and draft horse breeds of English and Spanish origin in North America in the late 18th to early 19th centuries (Regatieri et al., 2016). The described geographical distribution of haplotypes of clade N may reflect the predominant distribution of ancient haplotypes of this haplogroup in the western direction. It should be noted that clade N is not included in the haplogroup diversity of horses of the Sargarinsko-Alexeevskaya culture or mixed sites of both of the above-mentioned cultures under study.

The obtained data reflect the proximity of the mixed sites to the Sargarinsko-Alexeevskaya culture at the level of the gene pools of domestic horses, as well as differences in the mitogenomе genetic diversity of domestic horses of the Sargarinsko-Alexeevskaya and Irmen cultures, but how strong they were can only be understood by expanding the sample of Irmen culture horses. The phylogenetic closeness of the mitogenome haplotypes of the studied ancient and modern horses from different regions of the world most likely points to features in the migration directions of bearers of the Irmen and Sargarinsko-Alexeevskaya cultures after their collapse

## Conclusion

The study of mitochondrial genomes of ancient horses of the Sargarinsko-Alexeevskaya and Irmen cultures in the south of Western Siberia, mixed sites of these cultures located on the border between their areas, older cultures (Eluninskaya and Andronovo) in Western Siberia and Kazakhstan, and later cultures of Western Siberia and Mongolia revealed the presence of horse haplogroups of the most ancient of the studied cultures among horses of the Earle Iron Age Mountains and Forest-steppe Altai cultures. Our phylogenetic reconstructions are first to show the closeness of haplotypes of Mongolian horses of the Khereksur and “Deer Stone” and Andronovo cultures on the grounds of mitogenome data. They have also identified differences between the horses of the Sargarinsko- Alexeevskaya and Irmen cultures. The horses of the mixed sites with ceramics of both of the aforementioned cultures are closer in mitochondrial DNA to the horses of the Sargarinsko- Alexeevskaya culture, some haplotypes of which, in turn, fell into the haplogroup with the highest content of Akhal-Teke horses. Other haplotypes of this culture turn out to be genetically close to horse haplotypes of ancient indigenous breeds of Southern Europe and China. In contrast, the horses of the Irmen culture show a closer relationship to the horses of the native breeds of Northern Europe. The differences between the mitochondrial gene pool of horses of the Sargarinsko- Alexeevskaya and Irmen cultures may be associated with the origin of the studied horses of these cultures from different stock populations, as well as with the absence of a strong exchange of horses between them, which most likely correlates with the different histories of the cultures under consideration. The close mitochondrial relationship between Bronze Age Mongolian and Siberian Andronovo horses is most likely connected with the origin of the former from the latter. Further study of horses of the cultures under study is required to understand how strong the differences in their mitochondrial gene pools were.

## Conflict of interest

The authors declare no conflict of interest.
